# Listeners can extract meaning from non-linguistic infant vocalisations cross-culturally

**DOI:** 10.1038/srep41016

**Published:** 2017-01-25

**Authors:** Verena Kersken, Klaus Zuberbühler, Juan-Carlos Gomez

**Affiliations:** 1 School of Psychology, University of St Andrews, St Marys Quad, St Andrews, KY16 9JP, Scotland; 2 Budongo Conservation Field Station, P.O. Box 362, Masindi, Uganda; 3 Cognitive Science Centre, University of Neuchâtel, Rue Emile-Argand 11, 2000 Neuchâtel, Switzerland

## Abstract

We present empirical evidence showing that the acoustic properties of non-linguistic vocalisations produced by human infants in different cultures can be used cross-culturally by listeners to make inferences about the infant’s current behaviour. We recorded natural infant vocalisations in Scotland and Uganda in five social contexts; declarative pointing, giving an object, requesting an action, protesting, and requesting food. Using a playback paradigm, we tested parents and non-parents, who either had regular or no experience with young children, from Scotland and Uganda in their ability to match infant vocalisations of both cultures to their respective production contexts. All participants performed above chance, regardless of prior experience with infants or cultural background, with only minor differences between participant groups. Results suggest that acoustic variations in non-linguistic infant vocalisations transmit broad classes of information to listeners, even in the absence of additional cues from gesture or context, and that these cues may reflect universal properties similar to the ‘referential’ information discovered in non-human primate vocalisations.

In human speech, prosody changes the rhythm, stress, or intonation of an utterance and thereby conveys information beyond the semantic content of utterances, for example to indicate questions or make statements^1^. The prosodic features of speech also function to convey basic motivational and emotional states, for example, joy, disgust, sadness, or contempt, which can be recognised from the acoustic structure of the speech signal alone^2^. It has been suggested that this could be a human universal, as speakers of different languages can link differences intonations in a fictitious language with specific emotions^3^. Alongside speech and its associated prosodic patterns, humans produce vocal signals that have no direct linguistic content, such as grunts, cries, screams, laughter, or gasps. However, the communicative functions of these non-linguistic vocal signals, despite their ubiquity in everyday human interaction, have rarely been studied.

Some developmental studies suggest that infant non-linguistic sounds (e.g., crying) are primarily expressions of affect and emotional states^4^. A significant proportion of the vocal signals produced by human infants in their first six months of life are of this type and appear to function to express the infant’s primary needs, such as hunger or physical discomfort^5^. There is evidence that the acoustic properties of some of these sound types vary systematically with the context in which they are produced. For example, new-borns display acoustically different cry patterns when in pain as compared to when hungry^6^,^7^. Similarly, within the first three months, the acoustic properties of infant vocalisations emitted in a positive or negative emotional state vary systematically^7^,^8^. Furthermore, parents are able to distinguish these sounds, and make inferences about the infant’s emotional state on the basis of acoustic information alone^9^. Thus, parents listening to the vocalisations of infants recorded in different settings (when the infant was hungry, when their nappy needed changing, or when she was content), were able to classify the sounds they heard on a specially designed infant-state “barometer”^9^.

As the infant matures, these classes of sounds do not disappear but continue to be produced in co-existence with speech. For example, 4–8 month-olds consistently produce acoustically similar vocal patterns during toy interactions^10^, and 11–22-month-old infants produce acoustically consistent structures to express affect, indicate an event or object, or request help^11^,^12^. Whether these acoustic differences influence a listener’s behaviour is a question that received considerably less research interest. One study reported that different auditory cues that accompanied a video still frame of an infant led parents to make different judgements about the activity the infant is engaged in ref. 13. However, a recent study by Lindova *et al*. suggests that listeners can make judgements about emotional salience when listening to infant vocalisations, but fail to draw correct inferences about the production context^14^.

In 12-month old infants, declarative pointing to direct someone’s attention to an interesting event, and imperative pointing to request an object or action, are associated with acoustically different vocalisations^15^,^16^, and 9–18 month old infants produce acoustically distinct grunt variants linked to different situations, such as physical effort, attention to objects, and attention to people^17^,^18^. An interesting interpretation here has been that grunts are phylogenetically related to the grunts of non-human primates, and might facilitate the acquisition of referential words in humans^17^. While there is a wealth of research based on infants growing up in Western cultures, we have virtually no information about the use of non-linguistic vocal sounds in cross-cultural contexts and the type of information that they can transmit.

The aim of our study was to explore whether the non-linguistic vocalisations of human infants from different cultures convey referential information about situations and events independently of the linguistic and cultural background of participants. To address this, we investigated whether adult listeners from different cultures and with different degrees of experience with young children were able to interpret context-specific vocalisations produced by 11–18 month-old infants from their own or from another culture.

We hypothesized that a number of variables could potentially influence participants’ performance on this task: 1) the level of experience participants had with young children^19^, 2) whether the infant vocalisation was recorded in the listener’s own or another culture^20^, and 3) in what behavioural context the infant vocalisation is produced^14^. We explored the influence of these variables on people’s abilities to match infant vocalisations to their respective production context.

## Results

We employed a playback paradigm in which vocalisations from five different behavioural contexts (protesting, requesting an action, declarative pointing, giving an object, and requesting food – see Table 1) were played back to 102 listeners. To investigate the influence of culture, we tested listeners from Scotland and rural Uganda with vocalisations that we previously recorded from infants in both cultures. To investigate the role of experience, we tested parents, non-parents who regularly interacted with young infants, and non-parents who had no direct experience with infants (the latter only in Scotland – all our participants in Uganda had smaller siblings or shared a compound with families with young children).

Audio stimuli were presented to participants, and they were asked to choose from three descriptions (for example “infant wants food”, “infant points to a car” or “infant gives toy to a peer”), which they thought best fit the audio sample. Overall 40 audio stimuli from different individuals were presented, 20 of which were recorded in Scotland, and 20 in Uganda. We analysed the frequency of correct matchings between recording context of the audio sample and the description offered.

**Figure 1:**
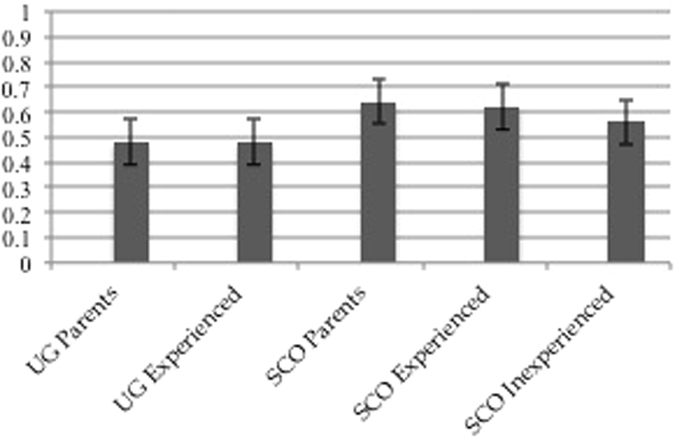
Proportion of correct responses in participant groups for each of the five stimuli categories.

In all of the five participant groups, participants scored a higher proportion of correct matchings of vocalisations and production contexts than would be expected by chance (i.e. 33% of correct responses, see Fig. 1 and Table 2) (one sample t-test on the proportion of correct responses: Scottish parents t[19] = 33.41, p = 0.0001, Scottish experienced t[16] = 52.59, p = 0.0001, Scottish inexperienced t[19] = 30.84, p = 0.0001, Ugandan parents t[19] = 22.67, p = 0.0001, Ugandan experienced t[20] = 27.32, p = 0.0001).

**Figure 2:**
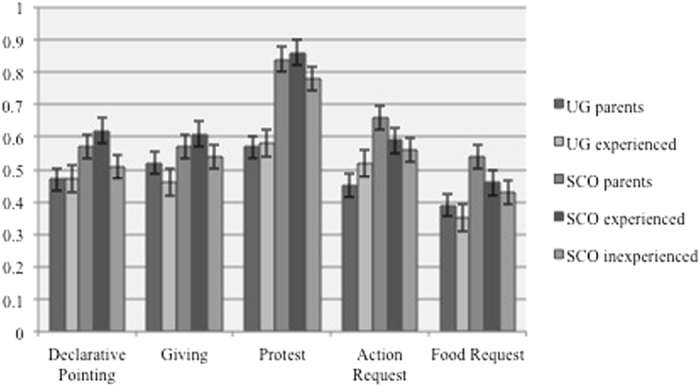
Proportion of correct responses for stimuli from both cultures for each participant group.

We chose a linear mixed-effects model fit by maximum likelihood, following Laird and Ware^21^. We tested a null-model (random factors: intercept and participant ID, nested within this variable were the following factors: participant origin, stimulus, context, participant group) against a full model that contained all predictor variables (fixed factors: participant origin, stimulus origin, context, participant group – see Table 3) to test if these would influence the participant’s ability to correctly match recordings to their production context. The full model was significantly better at predicting the participants’ success rate than the null-model (LRT: *χ*^*2*^_*1*_ = 61.66, p < 0.001). Context and participant group were significant predictors of the success rate, whereas stimulus origin was not a significant predictor (see Fig. 2 and Table 4).

## Discussion

In this playback study we show that Scottish and Ugandan adult participants were able to match audio samples of infant non-linguistic vocalisations to their corresponding behavioural contexts of emission, i.e., giving, declarative pointing, requesting actions, requesting food, and protesting. This was regardless of whether the vocalisations were recorded from infants in the listeners’ own or a different culture, and regardless of listeners’ previous experience with young infants.

The results of this study confirm and extend existing evidence that non-linguistic infant vocalisations contain information that can be picked up by receivers^11^,^13^,^14^,^16^. Importantly our study demonstrates for the first time that this information can be transmitted across cultures and is to some extent independent of the listener’s amount of experience with young infants.

An important issue is the nature of the information transmitted by the vocalizations. Although participants’ scores were significantly above chancel level, classification rates were far from perfect, around 50–60% (compared to 33% expected by chance, see Table 2). This suggests that the information content of the vocalisations is broad and semantically restricted. These broad referential functions were consistent across cultures, despite some evidence of fine-tuning by individual experience and cultural background.

In everyday situations, listeners are likely to encounter vocalisations alongside additional information provided by other communicative signals, such as gestures or facial expressions^22^,^23^, in addition to the situational context. These sources are likely to supplement the information contained in the vocalisations, and thereby increase the participants’ ability to recognise and classify situations, but our results show that the vocalizations themselves contain enough information to infer the situations above chance.

The relative independence from culture and experience of the ability to extract broad information from the infant vocal sounds supports the idea that the vocalisations we recorded were truly non-linguistic, as an early influence of native speech has been reported in vocalisations directly related to language acquisition: babbling sequences reflecting intonation or melodic patterns, and frequently used syllables, from the native language^20^,^24^,^25^.

The results of our study provide evidence that infants’ non-linguistic vocalisations transmit referential information about social events in which the caller is involved, regardless of upbringing. These referential functions may be comparable to what has been reported for non-human primates, and raise similar issues as to the nature of the referential functions and information involved. Contrary to previous findings that cross-cultural recognition of vocalisations is only accurate in relation to negative emotions or basic positive emotions^26^,^27^, the range of contexts recognised in our study is wider and richer, including information about subtle positive interactions like giving, showing, and cooperative requests of food and actions.

For many years, the default assumption, and still held by many, for primate vocalisations was that they purely reflect the caller’s states of arousal.

Very few studies, however, directly measure the role that arousal plays in the production of non-human primate vocal signals. It is possible that the production of these signals is, at least to some extent, affect-based, but listeners can still make inferences about the state of the world on the basis of this information^28^,^29^,^30^. The exact role affect plays in the production and comprehension of these signals needs further investigation, but the presence of affective information is not incompatible with fulfilling referential functions^31^.

The same question applies to the non-linguistic infant vocalisations presented here. It is possible that the contexts we described provoke affective reactions in the infants, and that the listeners infer the most likely situation to have provoked each vocalization based on its affective information. However unlike alarm calls in non-human primates, it might be more difficult to match all the vocalisations in this study to distinctive emotional states. While some vocalisations, for example ‘protests’, might be more easily explained as being primarily affect-driven and therefore more recognizable by their affective information, this might be more difficult in categories such as ‘declarative pointing’ or ‘giving’, that would require much subtler emotional distinctions, or maybe something akin to what in prosody is known as “paralinguistic attitudes”^1^. However, beyond the unresolved problem of what types of information are conveyed in the production and perception of these vocalisations, our results show that human listeners were able to make inferences about events in the world on the basis of the vocalisations alone.

Crucially, in our study all participants were able to recognise and classify the different classes of vocalisations above chance, regardless of their own or the signaller’s culture, suggesting that infant non-linguistic vocalisations are in this respect akin to those observed in non-human primates^32^,^33^. As with our findings, playbacks of primate calls provoke consistent behavioural reactions in receivers, despite individual differences in call structures, suggesting that directly or indirectly these signals convey information about the situation that provoked the vocalization. There is an on-going debate about the exact nature of the information contained in primate calls and in what sense it is or not referential in their production and comprehension^34^,^35^,^36^,^37^. Our results indicate that non-linguistic human vocalisations should be included in this debate.

## Methods

### Participants

102 adults volunteers took part in the study, 61 from Fife, Scotland, and 41 from the Masindi District, Uganda. The Scottish group consisted of 21 parents of infants older than two years, 20 participants with experience with children under the age of two years, and 20 with little or no experience. The Ugandan group consisted of 20 parents of infants older than two years, and 21 experienced participants (no non-experienced participants were found). The level of experience with infants was established through a self-report questionnaire, asking whether they had any children, and how old they were, or the open question of whether they had any experience with infants, and if so, to give examples of this (e.g. babysitting, job in nursery, younger relatives). If participants did not report any of these experiences, they were included in the group of “inexperienced non-parents”. We can, however, not exclude that this group gained some experience with infants through the media or more irregular contact with young children. Three experienced participants from Scotland were excluded, as they did not provide enough information about their experience in the questionnaire. In Scotland, all participants reported English as their first language or as being bilingual. All participants have completed secondary education, the majority either held a University degree, or where postgraduate students. Most participants came from a European middle-class background. Parents had between 1 and 3 children. In Uganda, participants were often multi-lingual, speaking Swahili, Alur or Acholi and all were able to read and understand written English. Formal education in Uganda is conducted in English, so the entire study was conducted in English for all participants. All Ugandan participants have completed at least primary education, some of the male participants also completed secondary education. All participants lived in rural villages in the Masindi district. In these communities the majority of people live in compounds shared with their large extended family and livestock. Some participants were professionals (teacher, shop-keeper), others were subsistence farmers who were occasionally employed. Parents had between 2 and 13 children.

### Playback Stimuli

Stimuli were selected from pre-recorded vocalisations of Scottish and Ugandan infants between the ages of 11 and 18 months in five different contexts (Table 1). The contexts were chosen because they occurred frequently in the infant’s everyday interactions in both cultures. Although we cannot completely rule out that some of these recordings carried traces of linguistic content, none of the calls revealed any resemblance to spoken words. Moreover, all infants were in the very early stages of speech development with a very small speech repertoire.

All audio stimuli were extracted from video recordings of natural interactions between the infants and their caregiver in a nursery environment (Scotland) or at home (Uganda). The video sequences were used to classify the stimuli according to context, type of interaction with persons and objects, and ongoing activities, using the broad categories of interactive behaviour listed in Table 1. Reliability of coding was ensured by asking two naïve coders to classify 15% of the video material from either culture into the five presented categories plus an additional ‘unknown’ category for cases that would not match any category. Inter-rater reliability was high (Cohen’s kappa = 0.96), suggesting that the context in which the sound was recorded could be identified unambiguously.

We then randomly selected eight audio clips (between 2 and 10 seconds long) from each of the five categories from our database, four produced by Scottish infants and four by Ugandan infants. The samples were produced by different infants. Using Adobe Audition we removed any background noise that could provide clues to the infant’s activity (e.g., hearing cutlery during food preparation). On some clips, the stimulus amplitude was enhanced to match other clips and to ensure that participants could hear the stimuli well. Otherwise the clips were not changed in any way.

### Experimental Set-up

In the experiment, participants were presented with 40 different recordings and asked, for each one, to select one description of infant behaviour that would best fit the audio clip from three options. We chose three options to allow participants a variety of choices without making too many demands on memory (participants had to remember the vocalization they were trying to match), or introducing possible confounds due to limited attention, misreading, and differences in reading skills between Ugandan and Scottish participants. All descriptions were taken from the transcripts of the original video episode that contained the infant call sample. The distracters were chosen randomly among descriptions from two different categories than the matching description. Distracters were counter-balanced to ensure an even representation of each category accompanying the target description. Descriptions were of the type ”infant sees more of a favourite food and requests some” or “infant gives an object to a friend”. Descriptions removed cues to the cultural background of the infant, for example, for the food context we removed culturally specific descriptions of food such as: ‘cheese’, ‘biscuits’ for Scotland, or ‘sweet potato’, ‘jackfruit’ for Uganda; or objects (Scotland: ‘soap bubbles’, ‘toys’; Uganda: ‘bucket’, ‘jerry cans’), or events of interest (Scotland: pointing at a boat; Uganda: pointing at goats).

Audio stimuli and the possible answers were presented on an Apple Macbook Pro computer in Scotland, and in Uganda on an Apple IPad 2. Before starting the experiment participants received instructions on how to work the technical equipment and what the experiment required of them, that is, to choose the description that they thought best fit the sound they heard. Two practice clips presented at the start served to familiarize participants with the procedure, who then completed 40 experimental trials. In each trial, participants were first presented with the empty screen and one of the audio clips. They could replay the sound ad libitum by operating a replay icon at the bottom of the page, until they chose to be presented with the three response options. Participants were asked to confirm their choice and were then presented the next trial. Participants were unaware that the audio samples were recorded from two different cultural backgrounds. Audio clips were presented in random order to avoid effects due to presentation order. Participants’ choices were recorded and whether or not these choices matched the recording context of the audio clip. Correct responses were coded when the participant’s choice matched the original recording context.

### Statistical Analysis

To investigate whether participants correctly matched a higher proportion of audio clips to their respective production context than expected by chance, we conducted a one-sample t-test on the proportion of correct response for each participant group.

To test whether participant’s home country (‘participant origin’), their experience with small children (‘participant group’), the country in which the vocalisation was recorded (‘stimulus origin’), or the recording context (‘context’) influenced participant’s ability to correctly match vocalisations to their respective production contexts, we ran a linear mixed-effects model fit by maximum likelihood, following Laird and Ware^21^. The statistical analysis was conducted in R, version 3.3.0 (R Core Team, 2016), and the nlme package (Pinheiro, Bates, DebRoy, Sarkar, and R Core Team). We tested whether the model predicted success in matching the playback stimuli to their respective production context and whether this is influenced by the fixed effects. The fixed effects, and their respective levels, are illustrated in Table 3. Our dependent variable was participant’s success in matching a recording to the correct production context.

To confirm model validity, we used variance inflation factors (VIF, Fox and Weisberg 2011), which verified that collinearity was not an issue (maximum VIF = 1.48). Using a Likelihood Ratio Test (LRT), we tested our full model against a null model comprising the intercept and random effect. We conducted an Analysis of Variance (ANOVA) on the baseline model to test how well different versions of the model describe the data and whether there are significant interactions between the fixed factors entered into the model. For the fixed factors with more than two levels (participant group and context), we conducted between-level comparisons (Table 5).

### Ethics

Participation was entirely voluntary and with no financial incentives. Participants were informed about the aims of the study and what their participation would entail. All participants gave their written consent to take part. After completion, participants were debriefed about the nature of the study. The study has been performed in accordance to the rules and regulations for research with human subjects of the University of St Andrews Teaching and Research Ethics Committee, and the Ugandan National Council for Science and Technology. Both bodies approved the study.

## Additional Information

**How to cite this article**: Kersken, V. *et al*. Listeners can extract meaning from non-linguistic infant vocalisations cross-culturally. *Sci. Rep.* **7**, 41016; doi: 10.1038/srep41016 (2017).

**Publisher's note:** Springer Nature remains neutral with regard to jurisdictional claims in published maps and institutional affiliations.

## Figures and Tables

**Table 1:** Descriptive contexts in which playback stimuli were recorded.

Category	Description
Giving	Infant gives object to peer or caregiver
Action request	Infant requests an action or object from a caregiver
Protest	Infant vocalises in reaction to an unpleasant event or action
Declarative Pointing	Infant points at an interesting object or event and vocalises
Food requests	Infant is in the presence of food and requests some

**Table 2:** Mean proportion of correct classifications for each participant group.

Participant group	Proportion of correct responses	Binomial Test p-values
UG parents	0.48	0.012
UG experienced non-parents	0.48	0.012
SCO parents	0.64	0.001
SCO experienced non-parents	0.62	0.001
SCO inexperienced non-parents	0.56	0.001

**Table 3:** Fixed effects and their levels.

Random/Fixed	Effect	Levels
Random	Participant ID	
Fixed	Stimulus Origin	Uganda Scotland
Fixed	Participant Origin	Uganda Scotland
Fixed	Context	Giving, Action Request, Protest, Declarative Pointing, Food Request
Fixed	Group	Parents, Experienced, Non-experienced

**Table 4:** Results of the GLMM testing factor that influence participants’ performance on the playback task.

	b	SE b	CI 95%	p-value
Intercept	0.892	0.049	0.797 to 0.988	
Stimulus Origin	−0.026	0.016	−0.056 to 0.005	0.099
Participant Origin	−0.148	0.018	−0.183 to −0.112	<0.001
Context	−0.014	0.006	−0.025 to −0.003	0.010
Group	−0.025	0.012	−0.048 to −0.001	0.039

**Table 5:** Between-level comparisons for multi-level factors (participant group and context).

Participant Group	Context	Mean	Standard Error	Standard Deviation
Parents	Food Request	0.537	0.029	0.499
	Action Request	0.533	0.029	0.499
	Protest	0.706	0.026	0.456
	Declarative Pointing	0.550	0.028	0.498
	Giving	0.399	0.028	0.490
Experienced	Food Request	0.518	0.027	0.500
	Action Request	0.556	0.027	0.497
	Protest	0.693	0.025	0.461
	Declarative Pointing	0.556	0.027	0.497
	Giving	0.500	0.027	0.500
Inexperienced	Food Request	0.506	0.039	0.501
	Action Request	0.543	0.039	0.499
	Protest	0.781	0.032	0.414
	Declarative Pointing	0.562	0.039	0.497
	Giving	0.425	0.039	0.495
